# A High-Density Nanoporous SERS Substrate Prepared by Facile One-Step Anodization for P-Hydroxybenzoic Acid Detection

**DOI:** 10.3390/s26134048

**Published:** 2026-06-25

**Authors:** Chin-An Ku, Chen-Kuei Chung

**Affiliations:** Department of Mechanical Engineering, National Cheng Kung University, Tainan 701, Taiwan

**Keywords:** anodic aluminum oxide, AAO, high-density porous substrate, surface-enhanced Raman scattering, SERS, p-hydroxybenzoic acid

## Abstract

Compared with mass spectrometry or high-performance liquid chromatography (HPLC), surface-enhanced Raman scattering (SERS) is a promising alternative technique for inspection of preservatives in food safety. However, conventional SERS substrates based on metallic nanoparticles commonly suffer from complicated fabrication processes, long processing times, and high costs. Therefore, we propose a high-density porous anodic aluminum oxide (AAO) substrate prepared by one-step anodization process combined with pore widening to increase number of SERS hotspots on template. Through a rapid one-step anodization process conducted at 25 °C, the processing time and efficiency are greatly improved compared to conventional low temperature of 0–10 °C and two-step anodization method. By lowering the anodization voltage to 20 V, a high-density porous substrate is achieved, effectively enhancing the SERS signal intensity. Furthermore, we demonstrated that SERS signal intensities are affected by multiple correlated structural factors and significantly improved by lower anodization voltage with pore widening. The analytical enhancement factor is calculated as 1.18 × 10^5^ to 1.44 × 10^7^ on an AAO substrate prepared at 20 V with pore-widening process for 1000 and 0.1 ppm p-hydroxybenzoic acid, respectively. For the preservative detection of p-hydroxybenzoic acid, a detection limit of 100 ppb is achieved by a high-density AAO substrate prepared at 20 V, which is far below the regulatory limit of 600 ppm.

## 1. Introduction

Surface-Enhanced Raman Scattering (SERS) has emerged as a powerful analytical technique for the detection of various chemical and biological substances due to its high sensitivity, molecular specificity, and rapid response. In recent years, SERS has been widely applied to the detection of food contaminants and additives, neurotransmitters, and heavy metal pollutants [1,2,3]. By significantly amplifying Raman signals through plasmonic nanostructures, SERS enables the identification of test substances. The development of simple and cost-effective fabrication methods for SERS substrates has further expanded its practical applicability. Advances in nanostructure engineering have enabled the production of highly sensitive substrates with lower detection limits. SERS has demonstrated great potential for food safety monitoring, biomedical diagnostics, and environmental pollution sensing. These advantages make SERS a promising technology for rapid, reliable, and on-site analysis in a wide range of applications.

Preservatives are widely used as food additives, and their detection is critically important for food safety with regulatory limit of 600 ppm. Among them, p-hydroxybenzoic acid is a common preservative that is widely added to foods [4,5] such as dried tofu, soy sauce, vinegar, beverages, and fruit surfaces. In recent years, studies have also indicated that p-hydroxybenzoic acid is related to plant analysis [6] and as a potential cancer biomarker [5,7], making its detection increasingly important. In general, p-hydroxybenzoic acid is commonly detected using methods such as mass spectrometry, high-performance liquid chromatography (HPLC) or differential pulse voltammetry [8,9,10,11]. However, these methods are complex, time-consuming, and require highly trained operators [12]. Although fluorometry methods have been proposed for rapid screening, the chemical synthesis of the substrate remains relatively time-consuming [13]. SERS is a promising alternative technique [14,15,16,17]. Conventional SERS technique is generally based on metallic nanoparticles (MNPs) [18,19,20,21,22,23], which suffer from complicated fabrication procedures, long processing time, and high costs. In addition, studies on SERS detection of p-hydroxybenzoic acid still mainly focus on the identification of characteristic peaks [24,25,26,27] and the measured concentration is approximately 10^−4^ M [28]. Although few studies have reported that p-hydroxybenzoic acid can be detected by SERS down to a very low concentration of 10^−11^ M, the result is based on calculation rather than direct experimental measurements [7]. Therefore, the development of SERS substrate with simple fabrication process for detection of p-hydroxybenzoic acid at lower concentration would provide significant benefits for practical applications.

In solid-state SERS substrates, anodic aluminum oxide (AAO) is a potential solution [29,30,31,32] for preservative detection. However, conventional two-step fabrication processes [33,34,35,36,37] with low temperature (0–10 °C) and identical voltage still suffer from long processing times or insufficient sensitivity. The test substance on most existing AAO substrates is based on common dyes, such as methylene blue, crystal violet or R6G. The studies on SERS substrates for detection of food additives in our daily life remain limited. Some AAO SERS substrates involve coating pre-prepared MNPs onto AAO substrates or fabricating 3D AAO nanostructures [30,38,39]. These methods increase the complexity of the fabrication process. In addition, the interpore distance of AAO is proportional to the anodization voltage, but most AAO-based SERS substrates reported, to date, are fabricated within a voltage range of 40–200 V [29,30,31,32,33,34,35,36,37]. As a result, the pore density of these AAO substrates is relatively low, and the potential benefits of high-density AAO pore structures have not been discussed. Therefore, developing high-density AAO substrates for p-hydroxybenzoic acid detection can not only clarify the relationship between AAO fabrication parameters and SERS signals, but also contribute to low-concentration detection of p-hydroxybenzoic acid. In our previous studies, we demonstrated that SERS enhancement on AAO surfaces is dominated by electromagnetic mechanism (EM) and originates from the pore peripherals (E_peri_), gaps (E_gap_), and tips (E_tip_) [40]. The EM and localized surface plasmon resonance (LSPR) effect of AAO-based SERS substrates has been verified by simulation [40]. In addition, we proposed AAO membrane detachment technique for 2D-3D nanostructures preparation on the bottom side of AAO membranes to further enhance SERS performance [41,42] for salicylic acid and sorbic acid [41,43] detection.

In this study, we propose a high-density two-dimensional porous AAO SERS template to achieve low-concentration detection of p-hydroxybenzoic acid. Compared with SERS detection using MNPs in solutions, we performed SERS detection with a solid-state AAO substrate by directly sputtering Ag to form a nanoporous film structure. Table 1 lists several SERS studies on detection of p-hydroxybenzoic acid [24,25,26,27,28,44,45] and AAO substrates for preservative detection [41,46] for comparison, to highlight the novelty of our approach. At present, SERS detection of p-hydroxybenzoic acid is mainly based on Ag nanoparticles, and the detection performance at low concentrations remains insufficient. In addition, SERS studies using AAO substrates are still largely focused on common dyes such as R6G or crystal violet [33,34,38,39], which are relatively easy for detection. The methodology of preparing SERS substrates by AAO for detection of preservatives remains relatively limited. Therefore, the development of high-density AAO substrates for p-hydroxybenzoic acid detection represents a promising research direction. By reducing anodization voltage to 20 V, pore density is increased and generates more hotspots around the pore peripherals. In addition, our previous studies have demonstrated that an increased total pore circumference (PC_total_) of AAO can effectively enhance sensing performance [43]. Accordingly, reducing the anodization voltage or applying pore-widening process can enhance performance of SERS substrates. Unlike conventional AAO fabrication with two-step anodization and removal processes lasting 6–24 h, our method completes substrate preparation within approximately 21–81 min and performs effective detections of p-hydroxybenzoic acid.

## 2. Materials and Methods

**Figure 1:**
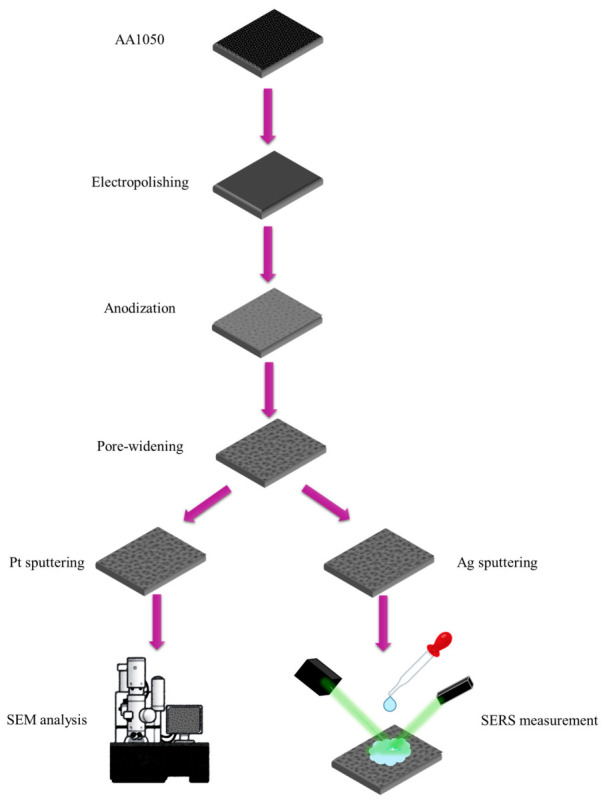
The experimental process flow of pore-widening AAO substrate and SERS measurement.

The experimental process flow is shown in Figure 1. First, 1050 aluminum alloy (AA1050) was cut into pieces of 2.5 × 2.5 cm. A two-step electropolishing process was performed using a mixture of perchloric acid and ethanol [47]. The electropolishing was carried out in perchloric acid and ethanol with volume ratio of 1:1 at 0 °C and 20 V for 1 min, followed by volume ratio of 1:4 at 0 °C and 20 V for 5 min. Subsequently, anodization was performed under three different conditions at 25 °C in 0.3 M oxalic acid by using the hybrid pulse anodization (HPA) method [43,48,49]. The anodization potentials were set at 20/−2 V or 40/−2 V with a duty ratio of 50% for 1 h, and 100/−4 V with a duty ratio of 20% for 10 min. The anodization process was achieved using a waveform generator (WF1947, NF corporation, Yokohama, Japan) with a voltage amplifier (PPS-2150, Jiehan, Taichung, Taiwan) and a two-electrode electrochemical system, with Pt mesh as the counter electrode and the AA1050 as the working electrode. The distance between Pt mesh and AA1050 is set as 10 cm, and the temperature is controlled by an external cooler (DANG YNG D-610, SHENG-CING, Kaosiung, Taiwan) to maintain at 25 ± 1 °C. After anodization, the AAO was immersed in 5 wt% phosphoric acid for pore widening at 35 °C for 10 min, followed by rinsing with deionized water.

After anodization or pore-widening process, a 12 nm Pt layer was coated on the AAO surface. The nanostructure of AAO is observed by high-resolution scanning electron microscope (HRFESEM, HITACHI, SU-5000, Tokyo, Japan) and analyzed by ImageJ software (ver. 1.53t). On the other hand, Ag sputtering was performed for 300 s (~20 nm) on AAO templates to complete the fabrication of the SERS substrate. A schematic diagram of the AAO SERS substrate is presented in Figure S1. For SERS measurements, p-hydroxybenzoic acid with concentrations ranging from 1000 to 0.1 ppm was used as the test substance. The uniformity was evaluated by a total of 10 measurement points of 1000 ppm p-hydroxybenzoic acid, including the first data point from the calibration curve together with nine additional points measured at the nine-grid positions on the substrate. The SERS measurement was performed by Raman spectrometer (GMDX, ProTrusTech, Tainan, Taiwan) with 532 nm laser. The laser power used during measurement was 10 mW, and the filter transmittance was set to 10%. Therefore, the actual power irradiated onto the substrate was approximately 1 mW. A 10× objective lens was used for measurements. The integration time was set to 1 s, and each spectrum was obtained by averaging data collected from three different positions. In addition, all SERS spectra were baseline-corrected and processed to remove noise and photoluminescence using the adaptive iteratively reweighted Penalized Least Squares (airPLS) method [50]. The calculation of analytical enhancement factor (AEF) is compared with the Raman signal on a Si wafer substrate.

## 3. Results and Discussions

**Figure 2:**
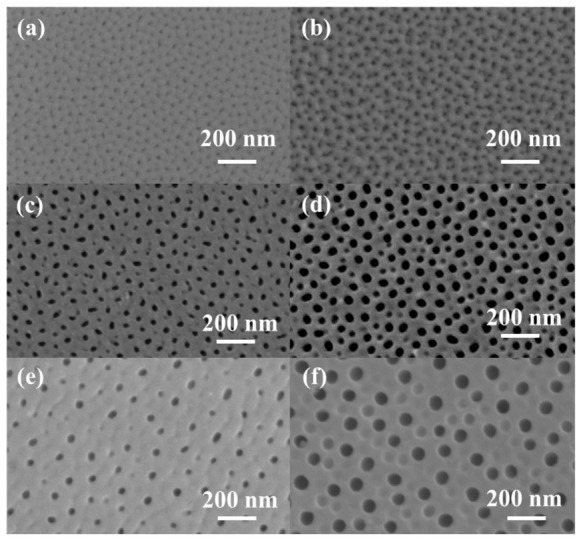
The SEM micrographs of AAO prepared at 25 °C in 0.3 M oxalic acid by (**a**) 20/−2 V, (**c**) 40/−2 V with 50% duty ratio for 1 h, and (**e**) 100/−4 V with 20% duty ratio for 10 min. (**b**,**d**,**f**) after pore widening in 5 wt% phosphoric acid at 35 °C for 10 min corresponding to (**a**,**c**,**e**), respectively.

Figure 2 shows SEM micrographs of AAO nanostructures prepared under different anodization voltages of 20/−2 V for 1 h (Figure 2a,b), 40/−2 V for 1 h (Figure 2c,d), and 100/−4 V for 10 min (Figure 2e,f). The cross-sectional SEM images of AAO are shown in Figure S2 for comparison. In Figure 2b,d,f, the AAO substrates are further immersed in 5 wt% phosphoric acid at 35 °C for 10 min for pore widening corresponding to Figure 2a,c,e, respectively. Compared with the conventional fabrication of AAO using high-purity aluminum through low-temperature two-step anodization, this technology employs a rapid, simple, and cost-effective method to fabricate AAO by one-step anodization of AA1050 at 25 °C. Although the anodization process was conducted at 25 °C, complete AAO nanopore structures without burning are preserved in all cases. This is attributed to the unique HPA method, which consists of a desired positive voltage followed by a smaller negative voltage, with a proper duty ratio. In anodization process, the negative voltage interval balances the reverse discharge caused by the capacitive characteristics of AAO, keeping the current close to zero and suppressing Joule heating. The HPA method not only enables the formation of complete AAO pores but also allows the reaction temperature to increase to 25 °C. This significantly overcomes the disadvantages of conventional anodization, which is typically limited to low temperatures and identical voltages with poor efficiency. Furthermore, with a proper duty ratio, the anodization potential is able to increase to 100/−4 V while still preserving a complete nanopore structure. Based on analysis of ImageJ software, the average pore diameters (D_p_) in Figure 2a–f are 19.2, 30.6, 27.9, 40.8, 40.6, and 68.9 nm, respectively. Since the interpore distance of AAO is proportional to the anodization voltage, a higher anodization potential results in lower pore density. The pore densities of AAO prepared at 20/−2 V, 40/−2 V, and 100/−4 V are 256, 161, and 63 pores/μm^2^, respectively. The related parameters are summarized and compared in Table 2.

**Figure 3:**
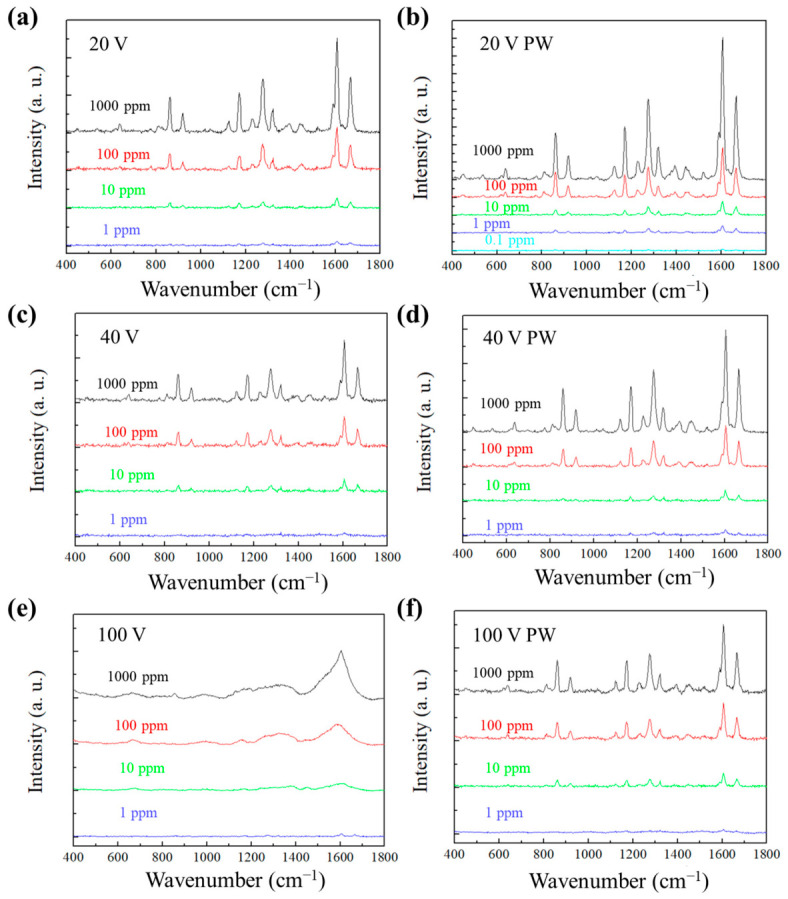
SERS spectra measured using AAO substrates fabricated at (**a**,**b**) 20/−2 V, (**c**,**d**) 40/−2 V, and (**e**,**f**) 100/−4 V. (**a**,**c**,**e**) were measured on AAO substrate before pore widening, whereas (**b**,**d**,**f**) were measured after phosphoric acid pore widening. The measurement concentration ranges from 1000 to 1 ppm in (**a**,**c**–**f**) and 1000 to 0.1 ppm in (**b**).

Figure 3 shows the SERS measurement results of p-hydroxybenzoic acid on different AAO substrates. The SERS spectra in Figure 3a–f are based on AAO nanoporous substrates shown in Figure 2a–f, respectively. The measured concentrations of p-hydroxybenzoic acid ranged from 1000 to 0.1 ppm and are represented by black, red, green, dark blue, and light blue lines, respectively. It is observed that p-hydroxybenzoic acid exhibits multiple characteristic SERS peaks, with the most prominent one located at the wavenumber range of 1606–1610 cm^−1^. This is in strong agreement with previously reported research results [28,51,52]. It is worth noting that only the AAO substrate fabricated at 20/−2 V with pore widening was able to produce an identifiable SERS signal for 0.1 ppm p-hydroxybenzoic acid. Therefore, the spectra for 0.1 ppm are shown in Figure 3b only. By comparing the SERS signal intensities in Figure 3a,c,e, it is found that the AAO substrate fabricated at a low voltage of 20 V exhibits the highest SERS intensity. This enhancement originates from the contribution of its high pore density. As reported in our previous research [40], the SERS enhancement of AAO substrates is mainly contributed by EM from pore peripherals, gaps, and sharp tips. However, under 2D AAO nanoporous structure and the same Ag sputtering conditions, the contribution of E_tip_ to various substrates is expected to be relatively minor. By comparing AAO nanostructures fabricated at different voltages in Figure 3a,c,e, it is evident that the AAO prepared at 20 V exhibits the highest pore density. Consequently, it generates the greatest number of hotspots from pore peripherals and gaps, which is clearly reflected in the enhancement of SERS signals. A comparison before and after pore widening shows that the pore-widening process effectively enhances the hotspot and EM around the pore peripherals and interpore gaps while maintaining the interpore distance, thereby further improving the sensing capability of the AAO substrate. As shown in Figure 3b, the AAO substrate fabricated at 20/−2 V with an additional pore-widening treatment achieves the best limit of detection (LOD), reaching 100 ppb for p-hydroxybenzoic acid detection, which is far below the regulatory limit of 600 ppm. Moreover, in the high-concentration range of 10–1000 ppm, the characteristic peaks are clearly distinguishable and easily identified. In contrast, the AAO fabricated at 100/−4 V has a lower pore density and, consequently, fewer hotspots. As a result, adjacent characteristic peaks tend to merge and become difficult to distinguish, as shown in Figure 3e.

**Figure 4:**
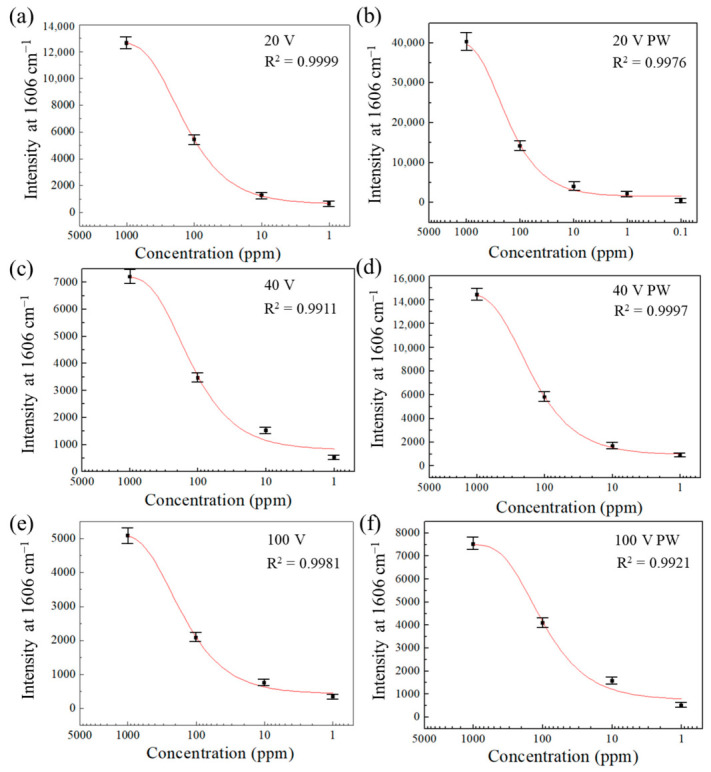
(**a**–**f**) Calibration curves of p-hydroxybenzoic acid corresponding to Figure 3a–f at 1606 cm^−1^. The measurement of p-hydroxybenzoic acid ranges from 1000 to 0.1 ppm on AAO substrate prepared at (**a**,**b**) 20/−2 V, (**c**,**d**) 40/−2 V, and (**e**,**f**) 100/−4 V, (**a**,**c**,**e**) before and (**b**,**d**,**f**) after pore widening. All R^2^ values exceed 0.99 with high predictability by natural function curve fitting. The average values and standard deviations are indicated by symbols and error bars, respectively.

Figure 4a–f presents the calibration curves plotted using peak intensities at 1606 cm^−1^ from SERS spectra shown in Figure 3a–f. The peak intensities at 1606 cm^−1^ for different concentrations of p-hydroxybenzoic acid are indicated by black dots, while the fitted natural function curves are shown as red lines. It is observed that all data in Figure 4a–f exhibit high predictability, with R^2^ values exceeding 0.99. Furthermore, in Figure 4b, the R^2^ value remains as high as 0.9976 even with five orders of concentrations measured. This demonstrates that the AAO SERS substrate fabricated at 20/−2 V with pore widening possesses excellent sensing capability. Furthermore, in Figure 4a–f, the measured signal intensities at 1606 cm^−1^ for 1000 ppm p-hydroxybenzoic acid are 12,628, 40,717, 7201, 14,958, 5091, and 7512, respectively. These values prove that high-density porous AAO substrates combined with pore widening effectively enhance E_peri_, E_gap_ and SERS signal intensity. Figure S3 shows the calibration curves of SERS intensities from p-hydroxybenzoic acid at (a) 1280 and (b) 1666 cm^−1^ measured on an AAO substrate prepared at 20/−2 V with pore widening for 10 min. The data is also collected from spectra in Figure 3b. The three characteristic peaks together clearly demonstrate that the detection of 0.1 ppm p-hydroxybenzoic acid can be achieved.

**Figure 5:**
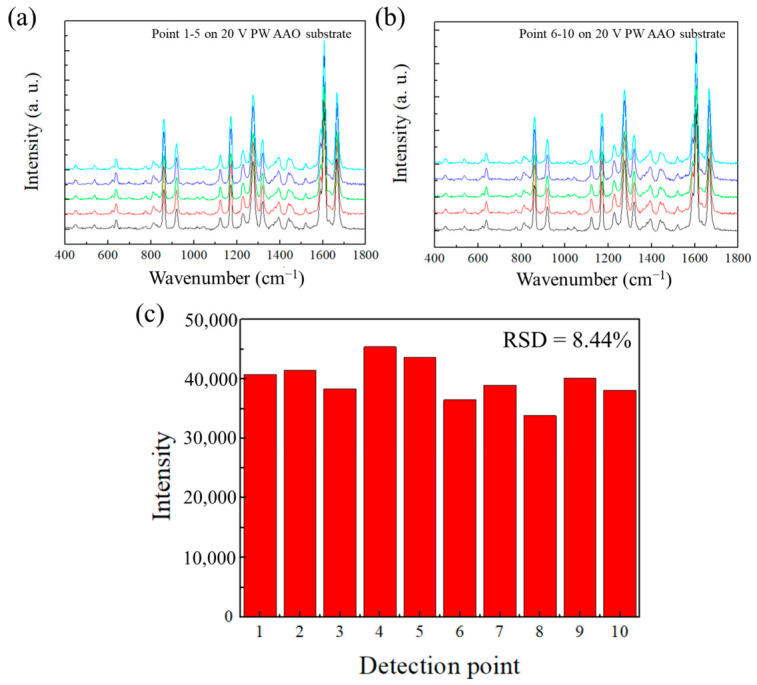
Measurements of 1000 ppm p-hydroxybenzoic acid on an AAO substrate prepared at 20/–2 V with pore widening: (**a**) points 1–5 and (**b**) points 6–10 from one data of Figure 3b at 1000 ppm together with the measured nine-grid positions across the substrate; (**c**) the RSD calculated from intensity at the wavenumber of 1606 cm^–1^ is 8.44%.

Figure 5 shows the uniformity of the AAO substrate prepared at 20/−2 V and after pore widening by 1000 ppm p-hydroxybenzoic acid detection. We calculated uniformity using a total of 10 spectra with spectra 1 from Figure 3b at 1000 ppm together with an additional nine measurements and plotted the results in Figure 5a,b. Nine additional measurements were taken at the nine-grid positions across the substrate. The relative standard deviation (RSD) was calculated to be 8.44%, as shown in Figure 5c. Figure S4 shows the second and third substrates fabricated under the same conditions, with five SERS measurement points collected on each substrate using 1000 ppm p-hydroxybenzoic acid. The spectra exhibit good consistency, and the RSD of the intensities at 1606 cm^−1^ for a total of 20 points is 8.54%, which is comparable to that of a single substrate.

**Figure 6:**
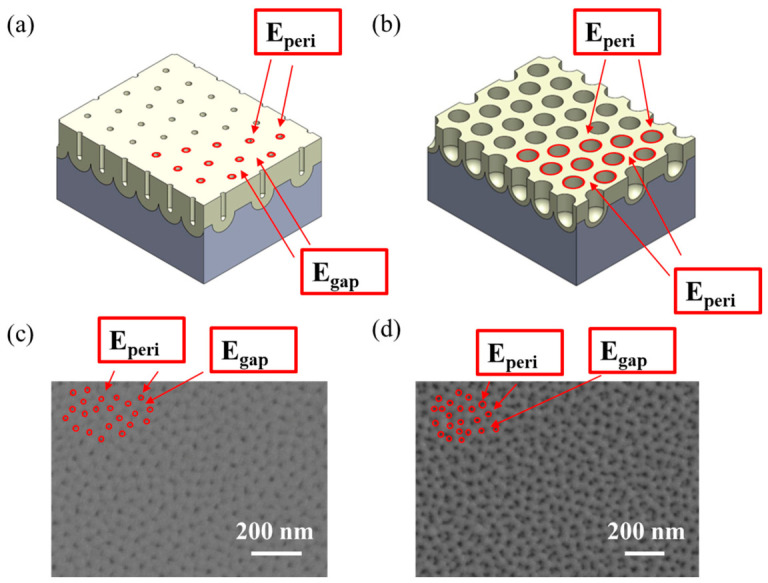
Schematic diagram of mechanism from AAO (**a**) before and (**b**) after pore widening. SEM images indicating hotspot regions (**c**) before pore widening and (**d**) after pore widening. After pore widening, the hotspots around AAO pore peripherals and interpore gaps significantly increase and improve SERS intensities.

Figure 6 presents the sensing mechanism of porous AAO as a SERS substrate. Figure 6a,b are the schematic diagram of AAO nanostructures before and after pore widening, respectively, with selected hotspot locations highlighted by red circles. Immediately after anodization, the AAO exhibits smaller D_p_ and consequently fewer hotspots. In contrast, the AAO after pore-widening process possesses larger pore sizes and, therefore, more hotspot regions, as indicated by E_peri_ in Figure 6b. Figure 6c,d are the actual SEM images before and after pore widening, respectively, with the hotspot locations on selected regions marked by red circles. Similarly, it is observed that the D_p_ of AAO before pore widening is smaller and, therefore, results in fewer effective hotspot sites. In contrast, after pore-widening process, more hotspot regions are generated, which effectively enhances the SERS signal. The electric field enhancement contributed by multiple correlated structural factors from AAO nanoporous SERS substrates originated from pore peripheral, interpore gaps, and sharp tips, and can be expressed by Formula (1) [40]:

(1) $$E_{total}=E_{peri}+E_{gap}+E_{tip}$$

In the two-dimensional AAO structures investigated in this study, all substrates were prepared under identical sputtering conditions. Although E_tip_ generated by small metal structures contributes to overall SERS intensity, the enhancement among different substrates is relatively minor. As a result, SERS enhancement mainly originates from pore peripheral and interpore gaps, which can be represented by Formulas (2) and (3) [40], respectively:

(2) $$E_{peri}=f_{1}\times E_{peri}^{\to }$$

(3) $$E_{gap}=f_{2}\times E_{gap}^{\to }$$

Here, $f_{1}$ and $f_{2}$ represent the coefficients of individual hotspots ($E_{peri}^{\to }\;or\;E_{gap}^{\to }$) associated with the pore peripheral and interpore gaps [40], respectively. $E_{\mathrm{peri}}$ is positively correlated with PC_total_, which is expressed in Formula (4) below [43]. $E_{\mathrm{gap}}$ is negatively correlated with the pore wall thickness [40].

(4) $${PC}_{total}=n\times D_{p}\times \pi$$

where n represents the number of pores per unit area and D_p_ denotes the pore diameter. According to the electric field enhancement mentioned above, SERS signal intensities are affected by multiple correlated structural factors and improved by lower anodization voltage and pore widening. Since AAO fabricated at lower voltages has smaller interpore distance, more significant enhancement is achieved under the same pore-widening conditions. In addition, the pore-widening process effectively reduces the interpore gap, further enhancing the SERS detection capability of the AAO substrate. Both reducing the anodization voltage and applying a pore-widening process can simultaneously enhance E_peri_ and E_gap_, thereby effectively improving the multiple correlated structural factors that improve the performance of SERS substrates. In addition, the SERS signal intensity of 1000 ppm p-hydroxybenzoic acid are plotted in Figure S5, showing a positive correlation with R^2^ values of 0.93 by natural function fitting. Therefore, preparing high-density porous AAO at low anodization voltages with Ag nanoporous film is an effective strategy to improve SERS detection performance. The EM of smooth AAO substrates is also simulated by COMSOL (ver. 6.3), as shown in Figure S6. In the COMSOL simulations, we employed an idealized pore geometry as the modeling structure. Since the morphology of sputtered metal films is difficult to describe using a specific and representative geometry, the AAO pore structure was used as the basis for simulations. The small surface structures from metal sputtering can indeed contribute to SERS enhancement and result in relatively higher electric field intensities than those predicted by simulations. We believe the simulated trends are still predictable and useful for analyzing the relationship between relative SERS intensity and substrate nanostructures. The simulation results are consistent with our experimental data, showing that the AAO substrate prepared at 20/−2 V with pore-widening process exhibits the highest electric field intensity. Furthermore, the hotspots are concentrated around pore peripherals and the evidenced proposed mechanism.

**Figure 7:**
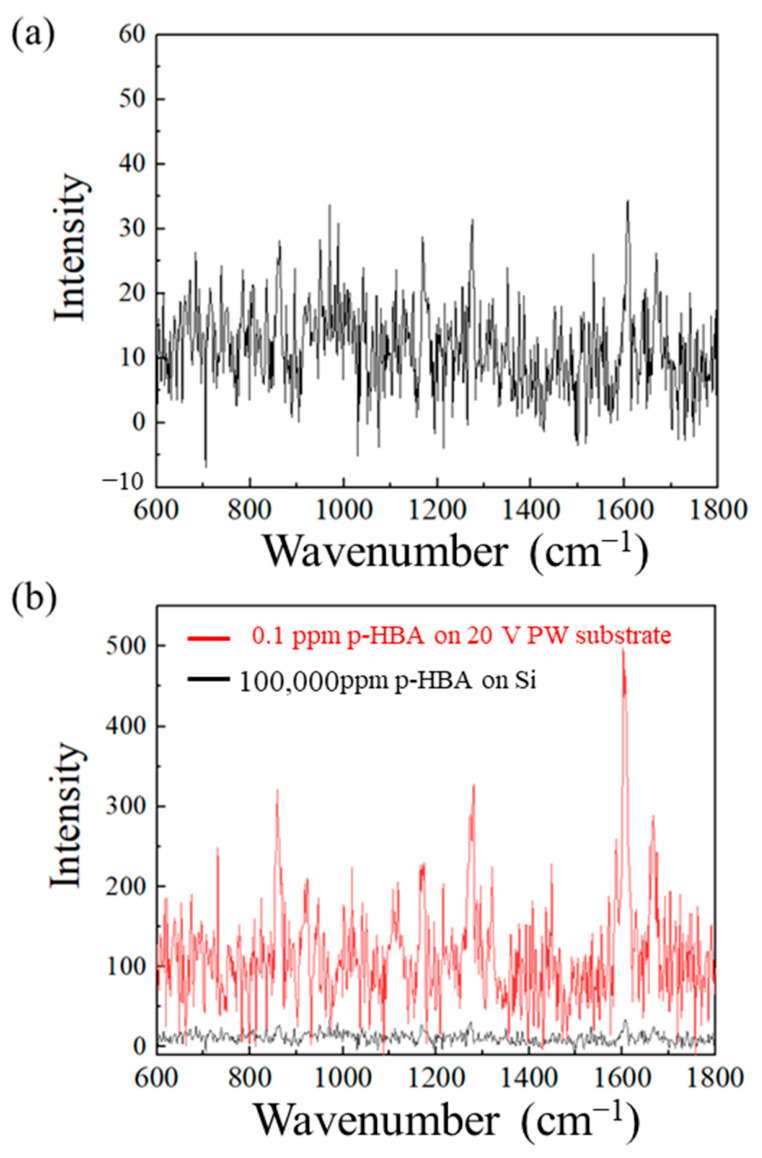
(**a**) Raman spectrum of 100,000 ppm p-hydroxybenzoic acid measured on a Si wafer. (**b**) SERS spectrum of 0.1 ppm p-hydroxybenzoic acid measured on AAO substrate prepared at 20/−2 V with pore-widening process.

AEF is another indicator of the sensing capability of solid-state SERS substrates. Figure 7a shows the Raman spectrum of 100,000 ppm p-hydroxybenzoic acid measured on a silicon wafer. A distinct peak can be observed at 1606 cm^−1^ with an intensity of approximately 34.5. Since the silicon substrate exhibits a strong characteristic peak at 520 cm^−1^, only the wavenumber range of 600–1800 cm^−1^ was selected for analysis. The AEF calculation is shown in Formula (5) below:

(5) $$AEF=\frac{C_{REF}}{C_{SERS}}\times \frac{I_{SERS}}{I_{REF}}$$

where C_SERS_ and C_REF_ are p-hydroxybenzoic acid concentrations of 1000 ppm on the AAO substrate prepared at 20/−2 V with pore widening and 100,000 ppm on the Si substrate. I_SERS_ and I_REF_ are the peak intensities at 1606 cm^−1^ of 1000 ppm p-hydroxybenzoic acid on the AAO substrate and 100,000 ppm p-hydroxybenzoic acid on Si, respectively. The AEF is calculated as 1.18 × 10^5^ with I_SERS_ = 40,717 and I_REF_ = 34.5. Figure 7b shows the comparison of SERS spectrum from 0.1 ppm p-hydroxybenzoic acid measured on the AAO substrate prepared by 20/−2 V with pore-widening process plotted as a red line (the same spectra in Figure 3b), while the Raman spectrum of 100,000 p-hydroxybenzoic acid measured on a silicon substrate is plotted as a black line (the same spectra in Figure 7a). Even with a concentration difference of six orders of magnitude, the pore-widened high-density AAO nanopore array substrate still exhibits a strong signal enhancement effect. In this case, the calculated AEF is 1.44 × 10^7^ with I_REF_ = 498.

Furthermore, several relevant SERS research for comparison are listed in Table 1. In general, the fabrication of AAO-based substrates relies on a two-step anodization process, often combined with additional Au or Ag nanoparticles deposition to form SERS substrates. This process is relatively time-consuming (>5 h), complex, and typically uses common dye for detection. On the other hand, studies on SERS detection of p-hydroxybenzoic acid are still limited, and most reported concentration ranges are relatively high (>10^−4^ M). In contrast, we propose a high-density AAO pore structure to prepare SERS substrates and successfully achieve LOD of 0.1 ppm for p-hydroxybenzoic acid detection. We propose a simple and rapid one-step AAO fabrication process at 25 °C and apply it to the less-explored detection of preservative. In addition, the morphology of high-density AAO porous substrates and their SERS enhancement effects are discussed in detail. This approach not only offers significant advantages in the fabrication process but also demonstrates a notable breakthrough in low-concentration detection of p-hydroxybenzoic acid.

## 4. Conclusions

We successfully developed a high-density porous AAO SERS substrate with Ag nanoporous film and applied it to the detection of p-hydroxybenzoic acid. Unlike a conventional AAO process, which is typically performed under low temperatures and using a two-step anodization process, we proposed a one-step anodization method at 25 °C to produce AAO with complete nanostructure. By applying the HPA method and modulating duty ratio, the influence of AAO substrates fabricated at different anodization voltages on SERS measurements can be effectively compared. Combined with a pore-widening treatment, the surface hotspots of an AAO substrate can be further increased, thereby enhancing the SERS signal intensity. Among different process parameters, AAO prepared at 20/−2 V and followed by pore-widening process exhibited the best performance by achieving AEF value between 1.18 × 10^5^ and 1.44 × 10^7^ and the lowest LOD of 100 ppb. The sensing performance is excellent and far below the regulatory limit of 600 ppm. Under natural function curve fitting, all R^2^ values exceed 0.99 and indicate that the signals exhibit high predictability. Furthermore, a comprehensive enhancement mechanism for AAO SERS substrates was established. We demonstrated that SERS signal intensities are affected by multiple correlated structural factors, and both reducing anodization voltage and applying pore-widening process can simultaneously enhance SERS performance. By employing low-voltage anodization and pore-widening process, the SERS signal intensity can be significantly improved, achieving the best LOD of 0.1 ppm in p-hydroxybenzoic acid detection.

## Figures and Tables

**Table 1:** Comparison of fabrication process and SERS performance on p-hydroxybenzoic acid detection on AAO substrate for preservative detection.

Ref.	Material/Morphology	Process/Time	Detection Substance	LOD
[24]	Polycrystalline gold rod/rough surface	Mechanically polishing and surface roughening/NA	p-hydroxybenzoic acid	0.02 M
[25]	Ag colloidal solution/nanoparticles	Chemical synthesis/>7 h	p-hydroxybenzoic acid	0.01 M
[26]	Ag nanoparticles on ITO/nanoparticles	Chemical synthesis/>8 h	p-hydroxybenzoic acid	0.01 M
[27]	Ag nanoparticles on filter paper/nanoparticles	Chemical synthesis/>7 h	p-hydroxybenzoic acid	0.33 (vol/vol)
[28]	Ag colloidal solution/nanoparticles	Laser ablation and chemical reduction method/NA	p-hydroxybenzoic acid	10^−4^ M
[44]	Ag colloidal solution/nanoparticles	Chemical synthesis/110 min	p-hydroxybenzoic acid	10^−2^ M
[45]	Ag colloidal solution/nanoparticles	Chemical synthesis/6 h	p-hydroxybenzoic acid	10^−3^ M
[46]	Ag nanoparticles in AAO pore/nanoparticles	Anodization and electrochemical plating/NA	Benzoic acid	1.5 mg/mL (1.2 × 10^−2^ M)
[41]	Ag nanoporous film on AAO membrane/porous membrane	Anodization and membrane detachment/3.3 h	Salicylic Acid	1 ppm
Ours	Ag nanoporous film on AAO substrate/porous substrate	Anodization/21–81 min	p-hydroxybenzoic acid	0.1 ppm (7.2 × 10^−7^ M)

**Table 2:** Comparison of pore density, pore diameter, perimeter, and total pore circumference of AAO substrates fabricated under different processing conditions and parameters.

Parameters	Number of Pores (unit/um^2^)	Perimeter (nm)	D_p_ (nm)	Total Pore Circumference (um/um^2^)
20 V	256	60.3	19.2	15.4
20 V PW	256	96.1	30.6	24.6
40 V	161	84.9	27.9	13.7
40 V PW	161	128.3	40.8	20.7
100 V	63	127.5	40.6	8.0
100 V PW	63	216.5	68.9	13.6

## Data Availability

The data are presented in the coauthors’ research results and schematic drawings, available on request.

## Supplementary Materials

The following supporting information can be downloaded at: https://www.mdpi.com/article/10.3390/s26134048/s1, Figure S1: The schematic image of AAO SERS substrate. Figure S2: Cross-sectional SEM images of AAO anodized at (a) 20/−2 V, (b) 40/−2 V for 1 h, and (c) 100/−4 V for 10 min. Figure S3: The calibration curve of SERS intensities from p-hydroxybenzoic acid at (a) 1280 and (b) 1666 cm−1 measured on AAO substrate prepared at 20/−2 V with pore widening for 10 min. Figure S4: (a) Five measurement points of 1000 ppm p-hydroxybenzoic acid on the second 20 V PW AAO substrate and (b) five measurement points on the third 20 V PW AAO substrate. (c) Comparison of the intensities at 1606 cm^−1^ obtained from a total of 20 measurement points of 1000 ppm p-hydroxybenzoic acid across three 20 V PW AAO substrates. Figure S5: (a) The relationship between PC_total_ of 2D AAO SERS substrate and SERS intensity from 1000 ppm p-hydroxybenzoic acid and (b) by natural function fitting. Figure S6: COMSOL simulation of AAO substrate prepared at (a) 20/−2 V, (c) 40/−2 V, and (e) 100/−4 V. (b,d,f) after pore widening from (a,c,e), respectively.
