# Supplementary material for: A High-Density Nanoporous SERS Substrate Prepared by Facile One-Step Anodization for P-Hydroxybenzoic Acid Detection
Source: Sensors (Basel). 2026 Jun 25;26(13):4048. doi: 10.3390/s26134048 (PMC13363926; doi:10.3390/s26134048)
Supplement: Supplementary file 1 [file sensors-26-04048-s001.zip › sensors-4310386-supplementary.pdf]

Supplementary Materials

Figure S1 presents a schematic diagram of the AAO SERS substrate, consisting of Ag, AAO, and aluminum from top to bottom. The aluminum alloy mainly serves as the substrate for AAO growth and has little direct relevance to SERS sensing. The pore morphology of the AAO layer ensures the formation of hotspots during Ag sputtering. The top Ag layer is closest to the target analyte and primarily contributes to the enhancement of the SERS signal.

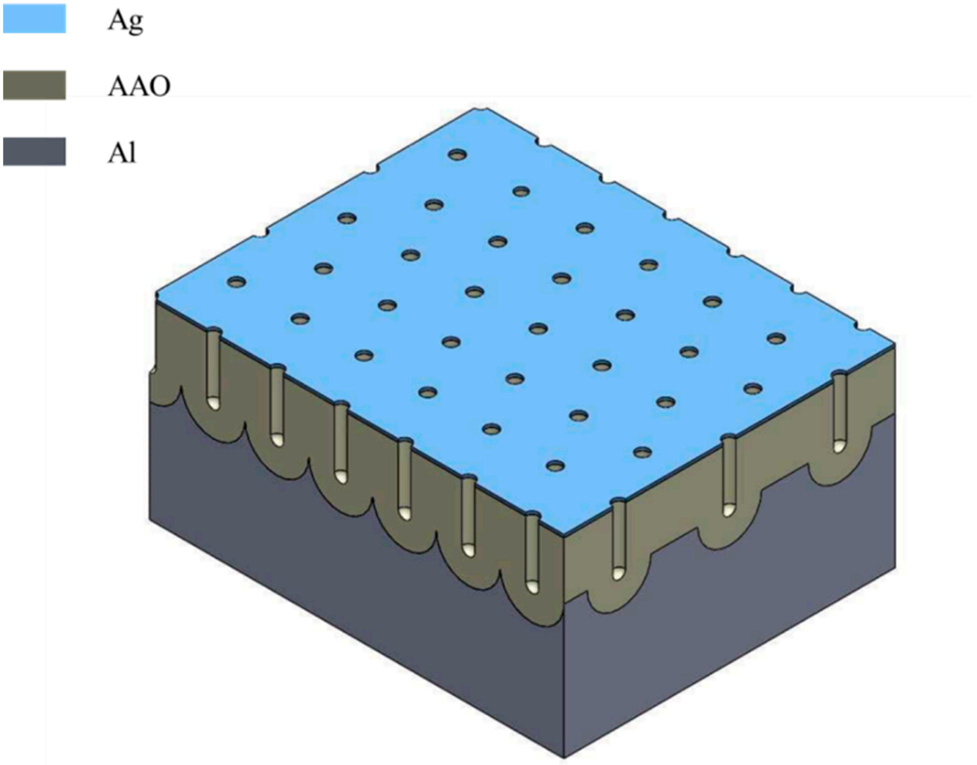

Figure S1. The schematic image of AAO SERS substrate.

Figure S2 shows the cross-sectional SEM images of AAO prepared in 0.3 M oxalic acid at 25 °C with anodization voltage at (a) 20/−2 V, (b) 40/−2 V for 1 h,, and (c) 100/−4 V for 10 min. The film thicknesses are 2.7, 5.8, and 16.8 μm, respectively.

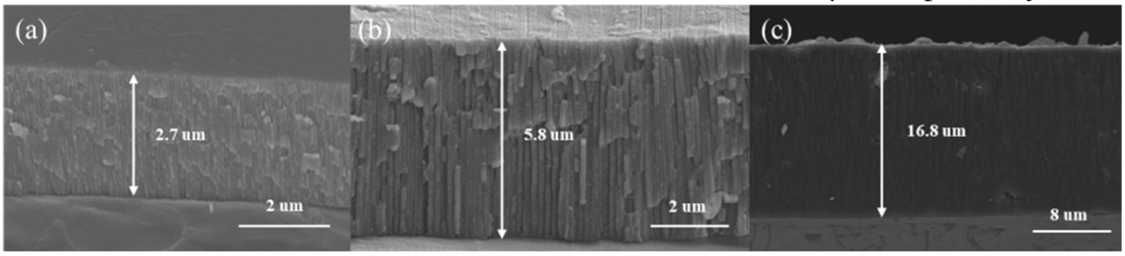

Figure S2. Cross-sectional SEM images of AAO anodized at (a) 20/−2 V, (b) 40/−2 V for 1 h, and (c) 100/−4 V for 10 min.

Figure S3 shows the calibration curves of SERS intensities from p-hydroxybenzoic acid at (a) 1280 and (b) 1666 cm^{-1} measured on AAO substrate prepared at 20/-2 V with pore widening for 10 min. The R^2 values exceed 0.95.

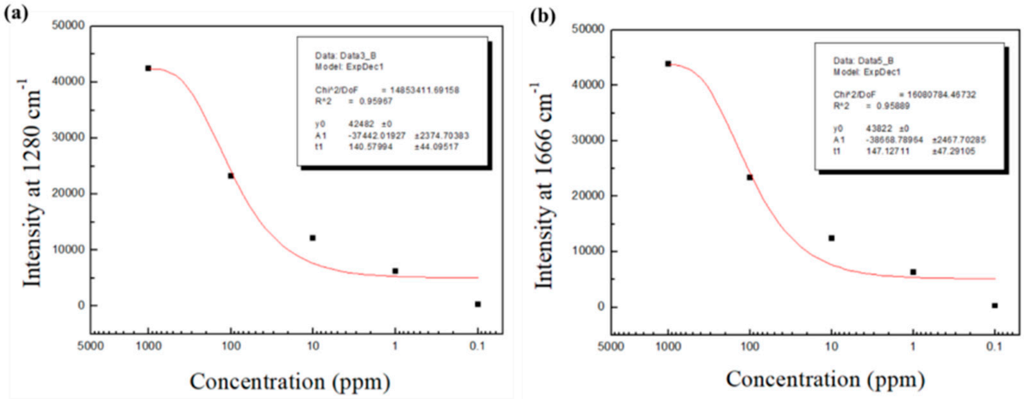

Figure S3. The calibration curve of SERS intensities from p-hydroxybenzoic acid at (a) 1280 and (b) 1666 cm^{-1} measured on AAO substrate prepared at 20/-2 V with pore widening for 10 min.

Figures S4(a) and (b) show the SERS spectra collected at top, left, bottom, right, and middle positions from 2nd and 3rd AAO substrate prepared at 20 V with pore widening for 10 min. Figure S4(c) compares the intensities at 1606 cm^{-1} from the total 20 points measured from 1st to 3rd substrate. The RSD is alculated about 8.54%.

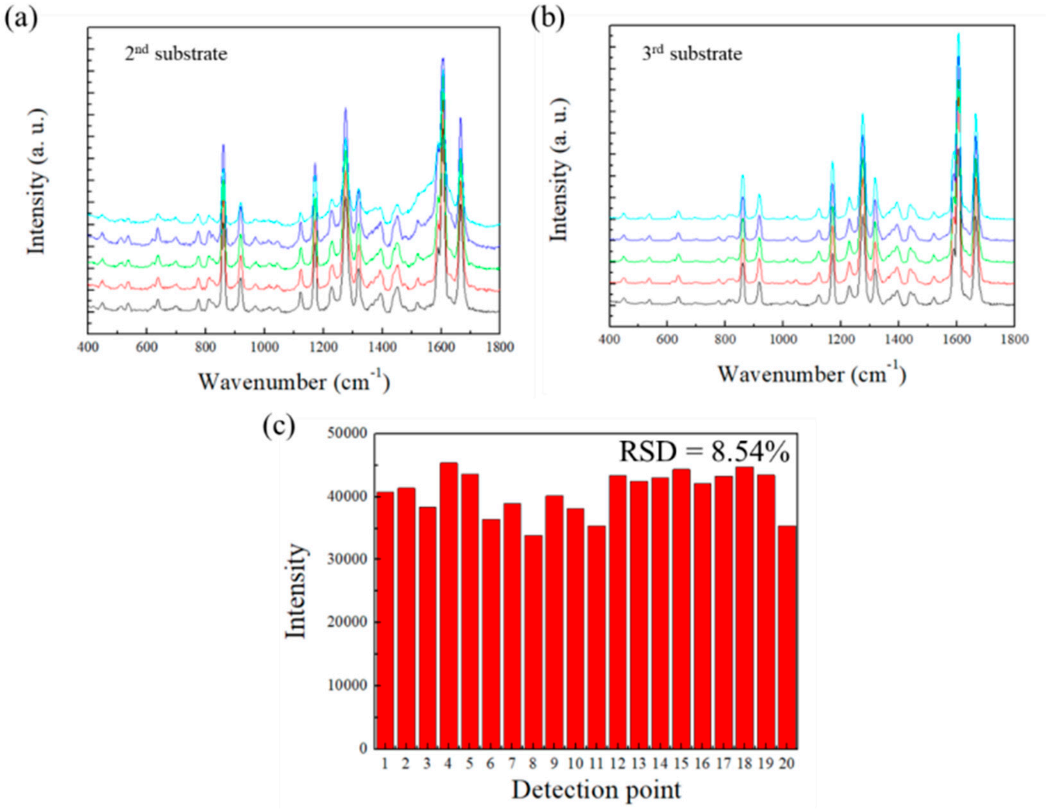

Figure S4. (a) Five measurement points of 1000 ppm p-hydroxybenzoic acid on the second 20 V PW AAO substrate and (b) five measurement points on the third 20 V PW AAO substrate. (c) Comparison of the intensities at 1606 cm^{-1} obtained from a total of 20 measurement points of 1000 ppm p-hydroxybenzoic acid across three 20 V PW AAO substrates.

Figure S5(a) shows the SERS signal intensity of 1000 ppm p-hydroxybenzoic acid and PC_{total} of substrates, showing a clear positive correlation and (b) with R^2 values of 0.93 by natural function fitting.

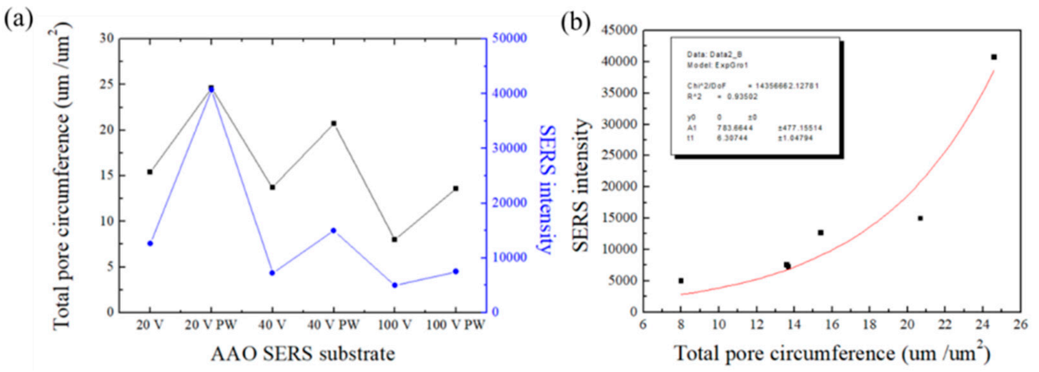

Figure S5. (a) The relationship between PC_{total} of 2D AAO SERS substrate and SERS intensity from 1000 ppm p-hydroxybenzoic acid and (b) by natural function fitting.

Figure S6 presents the simulated electrical field of AAO fabricated at (a) 20/-2 V, (c) 40/-2 V, and (e) 100/-4 V, and (b), (d), (f) further immersed in 5 wt% phosphoric acid at 35 °C for 10 min for pore widening. The COMSOL simulation is carried out by frequency-domain calculation with Drude-Lorentz model, with excitation of surface plasmons from a polarized plane wave. The parameters of material properties such as Ag, air, and Al_2O_3 follow the default in COMSOL software. We assumed a square computational domain (1 μm^2) that was significantly larger than the pore dimensions. A 532 nm laser was set to be normally incident on substrate, and the incident electromagnetic field was modeled as a plane wave. The thickness of the Ag layer was assumed to be 20 nm. Simulations with different structural parameters were performed by varying only the pore diameter and interpore distance while keeping all other conditions unchanged. The corresponding maximum electric field strengths are 28.1, 36.6, 22.1, 32.6, 12.3, and 20.4 V/m, respectively, which are consistent with the SERS signal intensity. The simulation results also evidenced that the electric field is indeed concentrated around pore peripherals and gaps, in agreement with our proposed mechanism.

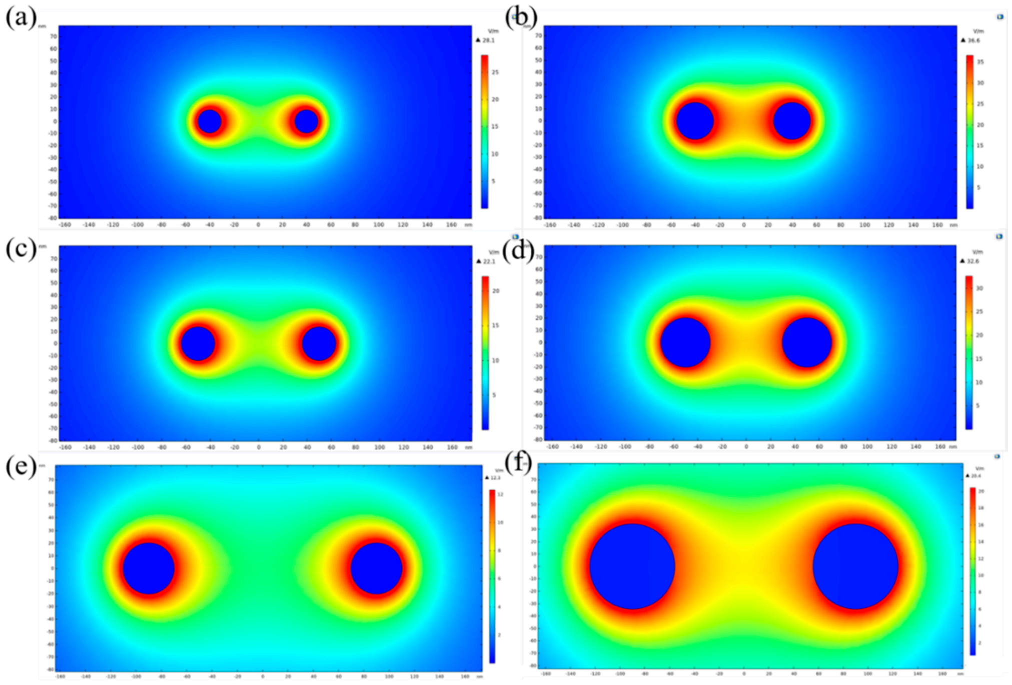

Figure S6. COMSOL simulation of AAO substrate prepared at (a) 20/-2 V, (c) 40/-2 V, and (e) 100/-4 V. (b)(d)(f) after pore widening from (a), (c) and (e), respectively.
